# Current Awareness on Comparative and Functional Genomics

**DOI:** 10.1002/1097-0061(200012)17:4<339::AID-YEA10>3.0.CO;2-X

**Published:** 2000


**Yeast**
*Yeast* 2000; **17:** 339–346

### Current awareness on comparative and functional genomics

### 1 Reviews & symposia

2000. Special issue: Pharmacogenomics. *Drug Dev Res* **49:** (1)

2000. Special issue: Parasite genomics. *Int J Parasitol* **30:** (4)

Alaiya AA, Franzen B, Auer G, Linder S. 2000. Karolinska Hosp, Canc Ctr, Unit Canc Proteomic, SE-17176 Stockholm, Sweden. Cancer proteomics: From identification of novel markers to creation of artificial learning models for tumor classification. *Electrophoresis* **21:** (6) 1210.

Bentley DR. 2000. Sanger Ctr, Wellcome Trust Genome Campus, Cambridge CB10 1SA, England. The human genome project: An overview. *Med Res Rev* **20:** (3) 189.

Bilban M, Head S, Desoye G, Quaranta V. 2000. Scripps Clin & Res Inst, Dept Cell Biol, 10550 Nth Torrey Pines Rd, La Jolla, Ca 92037, USA. DNA microarrays: A novel approach to investigate genomics in trophoblast invasion - A review. *Placenta* **21:** (Suppl A) S99.

Cash P. 2000. Univ Aberdeen, Dept Med Microbiol, Aberdeen AB27 2ZD, Scotland. Proteomics in medical microbiology. *Electrophoresis* **21:** (6) 1187.

Cordwell SJ, Nouwens AS, Verrills NM, Basseal DJ, Walsh BJ. 2000. Macquarie Univ, Australian Proteome Anal Facil, Nth Ryde, NSW 2109, Australia. Subproteomics based upon protein cellular location and relative solubilities in conjunction with composite two-dimensional electrophoresis gels. *Electrophoresis* **21:** (6) 1094.

Corthals GL, Wasinger VC, Hochstrasser DF, Sanchez JC. 2000. St Vincent's Hosp, Garvan Inst Med Res, 384 Victoria St, Sydney, NSW 2010, Australia. The dynamic range of protein expression: A challenge for proteomic research. *Electrophoresis* **21:** (6) 1104.

Cushman JC, Bohnert HJ. 2000. Oklahoma State Univ, Dept Biochem Mol Biol, Stillwater, Ok 74078, USA. Genomic approaches to plant stress tolerance. *Curr Opin Plant Biol* **3:** (2) 117.

Danchin A. 2000. Inst Pasteur, 28 rue Docteur Roux, FR-75724 Paris 15, France. A brief history of genome research and bioinformatics in France. *Bioinformatics* **16:** (1) 65.

De Benedetti VMG, Biglia N, Sismondi P, De Bortoli M*. 2000. *Ist Trattamento & Ric Canc, Str Prov 142 km 3.95, IT-10060 Candiolo, TO, Italy. DNA chips: The future of biomarkers. *Int J Biol Markers* **15:** (1) 1.

Diehn M, Alizadeh AA, Brown PO. 2000. Stanford Univ, Sch Med, Stanford, Ca 94305, USA. Examining the living genome in health and disease with DNA microarrays (Review). *JAMA* **283:** (17) 2298.

Dutt MJ, Lee KH*. 2000. *Cornell Univ, Sch Chem Engn, Ithaca, NY 14853, USA. Proteomic analysis (Review). *Curr Opin Biotechnol* **11:** (2) 176.

Emili AQ, Cagney G*. 2000. *Univ Washington, Dept Genet, Seattle, Wa 98195, USA. Large-scale functional analysis using peptide or protein arrays (Review). *Nat Biotechnol* **18:** (4) 393.

Freeman T. 2000. Sanger Ctr, Gene Express Grp, Hinxton Hall, Cambridge, England. High throughput gene expression screening: Its emerging role in drug discovery. *Med Res Rev* **20:** (3) 197.

Gevaert K, Vanderkerckhove J*. 2000. *State Univ Ghent, Flanders Interuniv Inst Biotechnol, Dept Biochem, BE-9000 Ghent, Belgium. Protein identification methods in proteomics. *Electrophoresis* **21:** (6) 1145.

Gorg A, Obermaier C, Boguth G, Harder A, Scheibe B, Wildgruber R, Weiss W. 2000. Tech Univ Munich, Inst Lebensmitteltechnol & Analyt Chem, FG Proteomik, DE-85350 Freising Weihenstephan, Germany. The current state of two-dimensional electrophoresis with immobilized pH gradients. *Electrophoresis* **21:** (6) 1037.

Hanash SM. 2000. Univ Michigan, Dept Pediat, Ann Arbor, Mi 48109, USA. Biomedical applications of two-dimensional electrophoresis using immobilized pH gradients: Current status. *Electrophoresis* **21:** (6) 1202.

Harris T. 2000. Struct GenomiX, 10505 Roselle St, San Diego, Ca 92121, USA. Genetics, genomics and drug discovery. *Med Res Rev* **20:** (3) 203.

Harry JL, Wilkins MR, Herbert BR, Packer NH, Gooley AA, Williams KL. 2000. Proteome Syst Ltd, Locked Bag 2073, Sydney, NSW 1670, Australia. Proteomics: Capacity *versus* utility. *Electrophoresis* **21:** (6) 1071.

Hsiao LL, Stears RL, Hong RL, Gullans SR*. 2000. *Harvard Univ, Brigham & Womens Hosp, Inst Med, Dept Med, Renal Div, 77 Ave Louis Pasteur, Boston, Ma 02115, USA. Prospective use of DNA microarrays for evaluating renal function and disease. *Curr Opin Nephrol Hypertens* **9:** (3) 253.

Jones HB. 2000. Stanford Univ, Dept Genet, Sch Med, Stanford, Ca 94305, USA. A review of statistical methods for genome mapping. *Int Statist Rev* **68:** (1) 5.

Kellner R. 2000. Merck KGaA, Preclin R&D, DE-64271 Darmstadt, Germany. Proteomics. Concepts and perspectives. *Fresenius J Anal Chem* **366:** (6-7) 517.

Kreider BL. 2000. Phylos Inc, 128 Spring St, Lexington, Ma 02421, USA. PROfusion(TM): Genetically tagged proteins for functional proteomics and beyond. *Med Res Rev* **20:** (3) 212.

Larsen MR, Roepstorff P*. 2000. *Odense Univ, Univ Sthn Denmark, Dept Mol Biol, DK-5230 Odense M, Denmark. Mass spectrometric identification of proteins and characterization of their post-translational modifications in proteome analysis. *Fresenius J Anal Chem* **366:** (6-7) 677.

Lee PS, Lee KH*. 2000. *Cornell Univ, Sch Chem Engn, Ithaca, NY 14853, USA. Genomic analysis (Review). *Curr Opin Biotechnol* **11:** (2) 171.

Lenz GR, Nash HM, Jindal S. 2000. Neogenesis, 840 Memorial Dr, Cambridge, Ma 02139, USA. Chemical ligands, genomics and drug discovery (Review). *Drug Discov Today* **5:** (4) 145.

Lopez MF. 2000. Proteomic Genomic Solution Inc, 22 Alpha Rd, Chelmsford, Ma 01824, USA. Better approaches to finding the needle in a haystack: Optimizing proteome analysis through automation. *Electrophoresis* **21:** (6) 1082.

Manabe T. 2000. Ehime Univ, Fac Sci, Dept Chem, Matsuyama, Ehime 790 8577, Japan. Combination of electrophoretic techniques for comprehensive analysis of complex protein systems. *Electrophoresis* **21:** (6) 1116.

McCarthy JJ, Hilfiker R. 2000. Millennium Predictive Med Inc, Cambridge, Ma 02139, USA. The use of single-nucleotide polymorphism maps in pharmacogenomics (Review). *Nat Biotechnol* **18:** (5) 505.

McClure MA. 2000. Montana State Univ, Dept Microbiol, Bozeman, Mt 59717, USA. The complexities of genome analysis, the retroid agent perspective (Review). *Bioinformatics* **16:** (2) 79.

McMaster G. 2000. Warner Lambert Parke Davis, Pharmaceut Res, Ann Arbor, Mi 48105, USA. The impact of genomics' technologies on

In order to keep subscribers up-to-date with the latest developments in their field, this current awareness service is provided by John Wiley & Sons and contains newly-published material on comparative and functional genomics. Each bibliography is divided into 16 sections. 1 Reviews & symposia; 2 General; 3 Large-scale sequencing and mapping; 4 Genome evolution; 5 Comparative genomics; 6 Gene families and regulons; 7 Pharmacogenomics; 8 Large-scale mutagenesis programmes; 9 Functional complementation; 10 Transcriptomics; 11 Proteomics; 12 Protein structural genomics; 13 Metabolomics; 14 Genomic approaches to development; 15 Technological advances; 16 Bioinformatics. Within each section, articles are listed in alphabetical order with respect to author. If, in the preceding period, no publications are located relevant to any one of these headings, that section will be omitted.

Copyright © 2000 John Wiley & Sons, Ltd.

pharmaceutical research. *Med Res Rev* **20:** (3) 187.

Merchant M, Weinberger SR*. 2000. *Ciphergen Biosyst Inc, 490 San Antonio Rd, Palo Alto, Ca 94306, USA. Recent advancements in surface-enhanced laser desorption/ionization-time of flight-mass spectrometry. *Electrophoresis* **21:** (6) 1164.

Michelmore R. 2000. Univ Calif, Dept Vegetable Crops, Davis, Ca 95616, USA. Genomic approaches to plant disease resistance. *Curr Opin Plant Biol* **3:** (2) 125.

Molloy MP. 2000. Univ Michigan, Dept Biol Chem, Ann Arbor, Mi 48109, USA. Two-dimensional electrophoresis of membrane proteins using immobilized pH gradients (Review). *Anal Biochem* **280:** (1) 1.

Mortimer RK. 2000. Univ Calif, Dept Mol & Cell Biol, 229 Stanley Hall, Berkeley, Ca 94720, USA. Evolution and variation of the yeast (*Saccharomyces*) genome (Review). *Genome Res* **10:** (4) 403.

Nelson RW, Nedelkov D, Tubbs KA. 2000. Intrinsic Bioprobes Inc, 2009 East 5th St, Suite 11, Tempe, Az 85281, USA. Biosensor chip mass spectrometry: A chip-based proteomics approach. *Electrophoresis* **21:** (6) 1155.

O'Connor CD, Adams P, Alefounder P, Farris M, Kinsella N, Li Y, Payot S, Skipp P. 2000. Univ Southampton, Dept Biochem, Basset Crescent East, Southampton SO16 7PX, England. The analysis of microbial proteomes: Strategies and data exploitation. *Electrophoresis* **21:** (6) 1178.

Parinov S, Sundaresan V. 2000. Natl Univ Singapore, Inst Mol Agrobiol, SG-117604 Singapore, Singapore. Functional genomics in *Arabidopsis*: Large-scale insertional mutagenesis complements the genome sequencing project (Review). *Curr Opin Biotechnol* **11:** (2) 157.

Patton WF. 2000. Molecular Probes Inc, Bioanalyt Assay Dev Grp, 4849 Pitchford Ave, Eugene, Or 97402, USA. Making blind robots see: The synergy between fluorescent dyes and imaging devices in automated proteomics (Review). *Biotechniques* **28:** (5) 944.

Patton WF. 2000. Address as above. A thousand points of light: The application of fluorescence detection technologies to two-dimensional gel electrophoresis and proteomics. *Electrophoresis* **21:** (6) 1123.

Pridmore RD, Crouzillat D, Walker C, Foley S, Zink R, Zwahlen MC, Brussow H, Petiard V, Mollet B. 2000. Nestec Ltd, Nestle Res Ctr, Vers Chez Les Blanc, CH-1000 Lausanne 26, Switzerland. Genomics, molecular genetics and the food industry. *J Biotechnol* **78:** (3) 251.

Richmond T, Somerville S. 2000. Carnegie Inst Washington, Dept Plant Biol, 290 Panama St, Stanford, Ca 94305, USA. Chasing the dream: Plant EST microarrays. *Curr Opin Plant Biol* **3:** (2) 108.

Rioux PP. 2000. Clin Pharmacogenomics & Pharmacoepidemiol, PMB 214, 405 Waltham St, Lexington, Ma 02421, USA. Clinical trials in pharmacogenetics and pharmacogenomics: Methods and applications. *Am J Health Syst Pharm* **57:** (9) 887.

Rohlff C. 2000. Oxford GlycoSci, 10 Quadrant, Abingdon Sci Pk, Abingdon OX14 3YS, England. Proteomics in molecular medicine: Applications in central nervous system disorders. *Electrophoresis* **21:** (6) 1227.

Rowley A, Choudhary JS, Marzioch M, Ward MA, Weir M, Solari RCE, Blackstock WP. 2000. Glaxo Wellcome R&D Ltd, Med Res Ctr, Gunnels Wood Rd, Stevenage SG1 2NY, England. Applications of protein mass spectrometry in cell biology. *Methods* **20:** (4) 383.

Santoni V, Molloy M, Rabilloud T*. 2000. *CEA, Dept Biol Mol & Struct, Lab Bioenerget Cellulaire & Pathol, 17 rue Martyrs, FR-38054 Grenoble 9, France. Membrane proteins and proteomics: Un amour impossible? *Electrophoresis* **21:** (6) 1054.

Sarto C, Binz PA, Mocarelli P. 2000. Univ Milan, Desio Hosp, Dept Clin Pathol, via Mazzini 1, IT-20033 Milan, Italy. Heat shock proteins in human cancer. *Electrophoresis* **21:** (6) 1218.

Sasaki T, Burr B. 2000. Natl Inst Agrobiol Resources, Rice Genome Res Program, 1-2 Kannondai 2 chome, Tsukuba, Ibaraki 305 8602, Japan. International Rice Genome Sequencing Project: The effort to completely sequence the rice genome. *Curr Opin Plant Biol* **3:** (2) 138.

Schaffer R, Landgraf J, Perez-Amador M, Wisman E. 2000. Michigan State Univ, East Lansing, Mi 48824, USA. Monitoring genome-wide expression in plants. *Curr Opin Biotechnol* **11:** (2) 162.

Searls DB. 2000. SmithKline Beecham Pharmaceut, 709 Swedeland Rd, POB 1539, King of Prussia, Pa 19406, USA. Using bioinformatics in gene and drug discovery (Review). *Drug Discov Today* **5:** (4) 135.

Sterky F, Lundeberg J. 2000. Royal Inst Technol, Dept Biotechnol, SE-10044 Stockholm, Sweden. Sequence analysis of genes and genomes (Review). *J Biotechnol* **76:** (1) 1.

Studholme DJ, Buck M. 2000. Univ London, Imperial Coll Sci Technol & Med, Dept Biol, Sir Alexander Fleming Bldg, London SW7 1AZ, England. The biology of enhancer-dependent transcriptional regulation in bacteria: Insights from genome sequences (Minireview). *FEMS Microbiol Lett* **186:** (1) 1.

Szymanski DB, Lloyd AM, Marks MD. 2000. Purdue Univ, Dept Agron, 1150 Lilly Hall Life Sci, West Lafayette, In 47907, USA. Progress in the molecular genetic analysis of trichome initiation and morphogenesis in *Arabidopsis* (Review). *Trends Plant Sci* **5:** (5) 214.

Talbot WS, Hopkins N. 2000. Stanford Univ, Dept Dev Biol, Stanford, Ca 94305, USA. Zebrafish mutations and functional analysis of the vertebrate genome (Review). *Genes Dev* **14:** (7) 755.

Van Hal NLW, Vorst O, Van Houwelingen AMML, Kok EJ, Peijnenburg A, Aharoni A, Van Tunen AJ, Keijer J. 2000. RIKILT, Gene Express & Detect Ctr, POB 230, NL-6700 AE Wageningen, The Netherlands. The application of DNA microarrays in gene expression analysis. *J Biotechnol* **78:** (3) 271.

Veraksa A, Del Campo M, McGinnis W*. 2000. *Univ Calif San Diego, Dept Biol, 9500 Gilman Dr, La Jolla, Ca 92093, USA. Developmental patterning genes and their conserved functions: From model organisms to humans (Minireview). *Mol Genet Metab* **69:** (2) 85.

Wambutt R, Murphy G, Volckaert G, Pohl T, Dusterhoft A, Stiekema W, Entian KD, Terryn N, Harris B, Ansorge W *et al*. 2000. c/o Bevan M, John Innes Ctr Plant Sci Res, Colney Lane, Norwich NR4 7UH, England. Progress in *Arabidopsis* genome sequencing and functional genomics. *J Biotechnol* **78:** (3) 281.

West DB, Iakougova O, Olsson C, Ross D, Ohmen J, Chatterjee A. 2000. Parke-Davis Lab Mol Genet, 1501 Harbor Bay Pkwy, Alameda, Ca 94502, USA. Mouse genetics/genomics: An effective approach for drug target discovery and validation. *Med Res Rev* **20:** (3) 216.

Xiang CC, Chen YD. 2000. UCLA, Microarray Core Facility, 695 Young Dr Sth, Los Angeles, Ca 90095, USA. cDNA microarray technology and its applications. *Biotechnol Advan* **18:** (1) 35.

### 3 Large-scale sequencing and mapping

Crooijmans RPMA, Vrebalov J, Dijkhof RJM, Van der Poel JJ, Groenen MAM. 2000. Univ Agr, Anim Breeding & Genet Grp, Postbox 338, NL-6700 AH Wageningen, The Netherlands. Two-dimensional screening of the Wageningen chicken BAC library. *Mamm Genome* **11:** (5) 360.

Huang M, Koh DCY, Weng LJ, Chang ML, Yap YK, Zhang L, Wong SM*. 2000. *Natl Univ Singapore, Dept Biol Sci, 10 Sci Dr 4, SG-117543 Singapore, Rep Singapore. Complete nucleotide sequence and genome organization of *Hibiscus* chlorotic ringspot virus, a new member of the genus *Carmovirus*: Evidence for the presence and expression of two novel open reading frames. *J Virol* **74:** (7) 3149.

Huang QH, Zhang CY*, Wang JF, Fu LZ, Wang N, Zhu Y, Huai JS, Zhang PW, Yu JS, Xu H. 2000. *Wuhan Univ, Inst Virol, CN-430072 Wuhan, Peoples Rep China. Construction of cDNA library, nucleotide sequence and analysis of entire genome of classical swine fever virus strain Shimen. *Chin Sci Bull* **45:** (4) 367.

Jabbari K, Bernardi G*. 2000. *Stn Zool Anton Dohrn, Lab Evolut Mol, Villa Comunale, IT-80121 Naples, Italy. The distribution of genes in the *Drosophila* genome. *Gene* **247:** (1-2) 287.

Kelly PD, Chu F, Woods IG, Ngo-Hazelett P, Cardozo T, Huang H, Kimm F, Liao LY, Yan YL, Zhou YY, Johnson SL, Abagyan R, Schier AF, Postlethwait JH, Talbot WS. 2000. Talbot WS, Stanford Univ, Sch Med, Dept Dev Biol, Stanford, Ca 94305, USA. Genetic linkage mapping of zebrafish genes and ESTs. *Genome Res* **10:** (4) 558.

Kitada K, Voigt B, Kondo Y, Serikawa T*. 2000. *Kyoto Univ, Inst Lab Anim, Sakyo ku, Yoshidakonoe cho, Kyoto 606 8501, Japan. An inte-

Copyright © 2000 John Wiley & Sons, Ltd. 

grated rat genome map based on genetic and cytogenetic data. *Exp Anim* **49:** (2) 119.

Lindblad-Toh K, Winchester E, Daly MJ, Wang DG, Hirschhorn JN, Laviolette JP, Ardlie K, Reich DE, Robinson E, Sklar P, Shah N, Thomas D, Fan JB, Gingeras T, Warrington J, Patil N, Hudson TJ, Lander ES. 2000. MIT, Whitehead Inst Biomed Res, Ctr Genome Res, Cambridge, Ma 02139, USA. Large-scale discovery and genotyping of single-nucleotide polymorphisms in the mouse. *Nat Genet* **24:** (4) 381.

Mathe C, Dehais P, Pavy N, Rombauts S, Van Montagu M, Rouze P*. 2000. *State Univ Ghent, Flanders Interuniv Inst Biotechnol, Dept Genet, INRA, Kl Ledganckstr 35, BE-9000 Ghent, Belgium. Gene prediction and gene classes in *Arabidopsis thaliana*. *J Biotechnol* **78:** (3) 293.

Murphy WJ, Sun S, Chen ZQ, Yuhki N, Hirschmann D, Menotti-Raymond M, O'Brien SJ. 2000. NCI, Frederick Canc Res & Dev Ctr, Lab Genomic Diversity, Frederick, Md 21702, USA. A radiation hybrid map of the cat genome: Implications for comparative mapping. *Genome Res* **10:** (5) 691.

Naruse K, Fukamachi S, Mitani H, Kondo M, Matsuoka T, Kondo S, Hanamura N, Morita Y, Hasegawa K, Nishigaki R, Shimada A, Wada H, Kusakabe T, Suzuki N, Kinoshita M, Kanamori A, Terado T, Kimura H, Nonaka M, Shima A. 2000. Univ Tokyo, Dept Biol Sci, Bunkyo ku, 7-3-1, Tokyo 113 0033, Japan. A detailed linkage map of medaka, *Oryzias latipes*: Comparative genomics and genome evolution. *Genetics* **154:** (4) 1773.

Narvel JM, Chu WC, Fehr WR*, Cregan PB, Shoemaker RC. 2000. *Iowa State Univ, USDA/ARS, Dept Agron, Ames, Ia 50011, USA. Development of multiplex sets of simple sequence repeat DNA markers covering the soybean genome. *Mol Breeding* **6:** (2) 175.

Nishigaki K, Saito A, Takashi H, Naimuddin M. 2000. Saitama Univ, Dept Funct Mat Sci, 255 Shimo Okubo, Urawa, Saitama 338 8570, Japan. Whole genome sequence-enabled prediction of sequences performed for random PCR products of *Escherichia coli*. *Nucleic Acids Res* **28:** (9) 1879.

Oelrichs RB, Lawson VA, Coates KM, Chatfield C, Deacon NJ, McPhee DA. 2000. Thai Res Cross Soc, AIDS Prog, 1871 Rama IV Rd, TH-10330 Bangkok, Thailand. Rapid full-length genomic sequencing of two cytopathically heterogeneous Australian primary HIV-1 isolates. *J Biomed Sci* **7:** (2) 128.

Rocha EPC, Guerdoux-Jamet P, Moszer I, Viari A, Danchin A*. 2000. *Inst Pasteur, 28 rue Docteur Roux, FR-75724 Paris 15, France. Implication of gene distribution in the bacterial chromosome for the bacterial cell factory. *J Biotechnol* **78:** (3) 209.

Ryazankina OI, Tumanova OY, Kolosova IV, Safronov PF, Kablova GV, Ryazankin IA, Shchelkunov SN. 2000. State Res Ctr Virol & Biotechnol Vector, Inst Mol Biol, RU-633159 Koltsov, Russia. Structural-functional organization of cowpox virus genome, strain GRI-90. I. Full genomic library. *Mol Biol-Engl Tr* **34:** (1) 141.

Slavov D, Hattori M, Sakaki Y, Rosenthal A, Shimizu N, Minoshima S, Kudoh J, Yaspo ML, Ramser J, Reinhardt R, Reimer C, Clancy K, Rynditch A, Gardiner K*. 2000. *Eleanor Roosevelt Inst Canc Res, 1899 Gaylord St, Denver, Co 80206, USA. Criteria for gene identification and features of genome organization: Analysis of 6.5 Mb of DNA sequence from human chromosome 21. *Gene* **247:** (1-2) 215.

Stewart P, Gaskell J, Cullen D*. 2000. *US Forest Serv, Forest Prod Lab, 1 Gifford Pinchot Dr, Madison, Wi 53705, USA. A homokaryotic derivative of a *Phanerochaete chrysosporium* strain and its use in genomic analysis of repetitive elements. *Appl Environ Microbiol* **66:** (4) 1629.

Strong WB, Nelson RG*. 2000. *San Francisco Gen Hosp, Div Infect Dis, San Francisco, Ca 94110, USA. Preliminary profile of the *Cryptosporidium parvum* genome: An expressed sequence tag and genome survey sequence analysis. *Mol Biochem Parasitol* **107:** (1) 1.

Szymanski M, Barciszewski J*. 2000. *Polish Acad Sci, Inst Bioorgan Chem, Noskowskiego 12-14, PL-61704 Poznan, Poland. Lessons from sequences genomes. Overlapping genes in *Methanococcus jannaschii*? *IUBMB Life* **49:** (2) 121.

Takami H, Horikoshi K. 2000. Japan Marine Sci & Technol Ctr, Deep Sea Microorganisms Res Grp, 2-15 Natushima, Yokosuka, Kanagawa 237 006, Japan. Analysis of the genome of an alkaliphilic *Bacillus* strain from an industrial point of view. *Extremophiles* **4:** (2) 99.

Zabarovsky ER, Gizatullin R, Podowski RM, Zabarovska VV, Xie L, Muravenko OV, Kozyrev S, Petrenko L, Skobeleva N, Li JF, Protopopov A, Kashuba V, Ernberg I, Winberg G, Wahlestedt C. 2000. Karolinska Inst, Ctr Genomics Res, Box 280, SE-17177 Stockholm, Sweden. *Not*I clones in the analysis of the human genome. *Nucleic Acids Res* **28:** (7) 1635.

Zhou XP, Xue CY, Chen Q, Qi YJ, Li DB. 2000. Zhejiang Univ, Inst Biotechnol, CN-310029 Hangzhou, Peoples Rep China. Complete nucleotide sequence and genome organization of tobacco mosaic virus isolated from *Vicia faba*. *Sci China C Life Sci* **43:** (2) 200.

### 4 Genome evolution

Chaw SM, Parkinson CL, Cheng YC, Vincent TM, Palmer JD*. 2000. *Indiana Univ, Dept Biol, 1001 East 3rd St, Bloomington, In 47405, USA. Seed plant phylogeny inferred from all three plant genomes: Monophyly of extant gymnosperms and origin of Gnetales from conifers. *Proc Natl Acad Sci U S A* **97:** (8) 4086.

Cloix C, Tutois S, Mathieu O, Cuvillier C, Espagnol MC, Picard G, Tourmente S*. 2000. *Univ Blaise Pascal, UMR 6547, Lab BIOMOVE, FR-63177 Clermont-Ferrand, France. Analysis of 5S rDNA arrays in *Arabidopsis thaliana*: Physical mapping and chromosome-specific polymorphisms. *Genome Res* **10:** (5) 679.

Forterre P, De la Tour CB, Philippe H, Duguet M. 2000. Univ Paris 11, Ctr Orsay, Inst Genet & Microbiol, Bat 400-409, FR-91405 Orsay, France. Reverse gyrase from hyperthermophiles: Probable transfer of a thermoadaptation trait from Archaea to bacteria. *Trends Genet* **16:** (4) 152.

Freeland SJ, Knight RD, Landweber LF, Hurst LD. 2000. Princeton Univ, Dept Ecol & Evolut, Princeton, NJ 08544, USA. Early fixation of an optimal genetic code. *Mol Biol Evol* **17:** (4) 511.

Graham DE, Overbeek R, Olsen GJ, Woese CR*. 2000. *Univ Illinois, Dept Microbiol, 601 Sth Goodwin Ave, Urbana, Il 61801, USA. An archaeal genomic signature. *Proc Natl Acad Sci U S A* **97:** (7) 3304.

Gregory TR, Hebert PDN, Kolasa J. 2000. Univ Guelph, Dept Zool, Guelph, Ontario, Canada N1G 2W1. Evolutionary implications of the relationship between genome size and body size in flatworms and copepods. *Heredity* **84:** (2) 201.

Jones TA, Redinbaugh MG, Zhang Y. 1999. Utah State Univ, USDA/ARS, Forage & Range Res Lab, Logan, Ut 84322, USA. The western wheatgrass chloroplast genome originates in *Pseudoroegneria*. *Crop Sci* **40:** (1) 43.

Kiewitz C, Tummler B. 2000. Hannover Med Sch, Klin Forschergrp, Carl Neuberg Str 1, DE-30625 Hannover, Germany. Sequence diversity of *Pseudomonas aeruginosa*: Impact on population structure and genome evolution. *J Bacteriol* **182:** (11) 3125.

Peterson-Burch BD, Wright DA, Laten HM, Voytas DF. 2000. Iowa State Univ, Dept Zool & Genet, 2208 Mol Biol Bldg, Ames, Ia 50011, USA. Retroviruses in plants? *Trends Genet* **16:** (4) 151.

Tillier ERM, Collins RA. 2000. Univ Toronto, Dept Mol & Med Genet, 1 Kings Coll Circle, Toronto, Ontario, Canada M5S 1A8. The contribution of replication orientation, gene direction, and signal sequences to base-composition asymmetries in bacterial genomes. *J Mol Evol* **50:** (3) 249.

Torti C, Gomulski LM, Moralli D, Raimondi E, Robertson HM, Capy P, Gaspari G, Malacrida AR*. 2000. *Univ Pavia, Dept Anim Biol, Piazza Botta 9, IT-27100 Pavia, Italy. Evolution of different subfamilies of *mariner* elements within the medfly genome inferred from abundance and chromosomal distribution. *Chromosoma* **108:** (8) 523.

Wang XQ, Tank DC, Sang T*. 2000. *Michigan State Univ, Dept Bot & Plant Pathol, East Lansing, Mi 48824, USA. Phylogeny and divergence times in Pinaceae: Evidence from three genomes. *Mol Biol Evol* **17:** (5) 773.

Worning P, Jensen LJ, Nelson KE, Brunak S, Ussery DW*. 2000. *Tech University, Ctr Biol Sequence Anal, DK-2800 Lyngby, Denmark. Structural analysis of DNA sequence: Evidence for lateral gene trans-

Copyright © 2000 John Wiley & Sons, Ltd. 

fer in *Thermotoga maritima*. *Nucleic Acids Res* **28:** (3) 706.

Yoshida T, Obata N, Oosawa K*. 2000. *Nagoya Univ, Grad Sch Math, Chikusa ku, Nagoya, Aichi 464 8602, Japan. Color-coding reveals tandem repeats in the *Escherichia coli* genome. *J Mol Biol* **298:** (3) 343.

### 5 Comparative genomics

Bouck JB, Metzker ML, Gibbs RA. 2000. Baylor Coll Med, Human Genome Sequencing Ctr, Dept Mol & Human Genet, Houston, Tx 77030, USA. Shotgun sample sequence comparisons between mouse and human genomes. *Nat Genet* **25:** (1) 31.

Bowe LM, Coat G, De Pamphilis CW*. 2000. *Penn State Univ, Inst Mol Evolut Genet, Dept Biol, University Park, Pa 16802, USA. Phylogeny of seed plants based on all three genomic compartments: Extant gymnosperms are monophyletic and Gnetales' closest relatives are conifers. *Proc Natl Acad Sci U S A* **97:** (8) 4092.

Braun EL, Halpern AL*, Nelson MA, Natvig DO. 2000. *Celera Genomics, Rockville, Md 20850, USA. Large-scale comparison of fungal sequence information: Mechanisms of innovation in *Neurospora crassa* and gene loss in *Saccharomyces cerevisiae*. *Genome Res* **10:** (4) 416.

Culetto E, Sattelle DB*. 2000. *Univ Oxford, Dept Human Anat & Genet, MRC Funct Genet Unit, Sth Parks Rd, Oxford OX1 3QX, England. A role for *Caenorhabditis elegans* in understanding the function and interactions of human disease genes. *Hum Mol Genet* **9:** (6) 869.

Grant D, Cregan P, Shoemaker RC. 2000. USDA/ARS, Iowa State Univ, Dept Agron, Ames, Ia 50011, USA. Genome organization in dicots: Genome duplication in *Arabidopsis* and synteny between soybean and *Arabidopsis*. *Proc Natl Acad Sci U S A* **97:** (8) 4168.

Kaisaki PJ, Rouard M, Danoy PAC, Wallis RH, Collins SC, Rice M, Levy ER, Lathrop M, Bihoreau MT, Gauguier D*. 2000. *Univ Oxford, Wellcome Trust Ctr Human Genet, Roosevelt Dr, Oxford OX3 7BN, England. Detailed comparative gene map of rat chromosome 1 with mouse and human genomes and physical mapping of an evolutionary chromosomal breakpoint. *Genomics* **64:** (1) 32.

Novichkov PS, Gelfand MS, Mironov AA. 2000. State Res Ctr Gosniigenet, RU-113545 Moscow, Russia. Prediction of the exon-intron structure by comparison of genomic sequences. *Mol Biol-Engl Tr* **34:** (2) 200.

Pan QL, Liu YS, Budai-Hadrian O, Sela M, Carmel-Goren L, Zamir D, Fluhr R*. 2000. *Weizmann Inst Sci, Dept Plant Sci, POB 26, IL-76100 Rehovot, Israel. Comparative genetics of nucleotide binding site-leucine rich repeat resistance gene homologues in the genomes of two dicotyledons: Tomato and *Arabidopsis*. *Genetics* **155:** (1) 309.

Thomas JW, Summers TJ, Lee-Lin SQ, Maduro VVB, Idol JR, Mastrian SD, Ryan JF, Jamison DC, Green ED*. 2000. *NIH/Natl Human Genome Res Inst, Genome Technol Branch, Bethesda, Md 20892, USA. Comparative genome mapping in the sequence-based era: Early experience with human chromosome 7. *Genome Res* **10:** (5) 624.

Wang N, Chen RS*, Wang YX. 2000. *Chinese Acad Sci, Inst Biophys, Prot Engn Lab, CN-100101 Beijing, Peoples Rep China. Conservation of ribosomal protein gene ordering in 16 complete genomes. *Sci China C Life Sci* **43:** (2) 120.

### 6 Gene families and regulons

Aubourg S, Boudet N, Kreis M, Lecharny A*. 2000. *Univ Paris 11, CNRS, UMR 8618, Inst Biotechnol Plantes, Bat 630, FR-91405 Orsay, France. In *Arabidopsis thaliana*, 1% of the genome codes for a novel protein family unique to plants. *Plant Mol Biol* **42:** (4) 603.

Gupta A. 1999. Univ Illinois, Coll Med, Dept Microbiol & Immunol, M/C 790, Room 703, 835 Sth Wolcott Ave, Chicago, Il 60612, USA. RT-PCR: Characterization of long multi-gene operons and multiple transcript gene clusters in bacteria. *Biotechniques* **27:** (5) 966.

Littleton JT, Ganetzky B. 2000. MIT, Ctr Learning & Memory, 77 Massachusetts Ave, Cambridge, Ma 02139, USA. Ion channels and synaptic organization: Analysis of the *Drosophila* genome. *Neuron* **26:** (1) 35.

Lloyd TE, Verstreken P, Ostrin EJ, Phillippi A, Lichtarge O, Bellen HJ*. 2000. *Baylor Coll Med, Dept Mol & Cellular Biol, Houston, Tx 77030, USA. A genome-wide search for synaptic vesicle cycle proteins in *Drosophila*. *Neuron* **26:** (1) 45.

Mekhedov S, Dellarduya OM, Ohlrogge J*. 2000. *Michigan State Univ, Dept Bot & Plant Pathol, East Lansing, Mi 48824, USA. Toward a functional catalog of the plant genome. A survey of genes for lipid biosynthesis. *Plant Physiol* **122:** (2) 389.

Ratner VA, Yudanin AY. 2000. Russian Acad Sci, Inst Cytol & Genet, RU-630090 Novosibirsk, Russia. Truncation family selection and non systematic inbreeding lead to rapid fixation of patterns of mobile genetic elements in a simulation model. *Russ J Genet* **36:** (3) 325.

Throup JP, Koretke KK, Bryant AP, Ingraham KA, Chalker AF, Ge YG, Marra A, Wallis NG, Brown JR, Holmes DJ, Rosenberg M, Burnham MKR*. 2000. *SmithKline Beecham Pharmaceut Res & Dev, Antiinfect Res, 1250 Sth Collegeville Rd, Collegeville, Pa 19426, USA. A genomic analysis of two-component signal transduction in *Streptococcus pneumoniae*. *Mol Microbiol* **35:** (3) 566.

Upton C. 2000. Univ Victoria, Dept Biochem & Microbiol, POB 3055, Victoria, Brit Columbia, Canada V8W 3P6. Screening predicted coding regions in poxvirus genomes. *Virus Genes* **20:** (2) 159.

Vanet A, Marsan L, Labigne A, Sagot MF*. 2000. *Inst Pasteur, Serv Informat Sci, 28 rue Dr Roux, FR-75724 Paris, France. Inferring regulatory elements from a whole genome. An analysis of *Helicobacter pylori* $\delta^{80}$ family of promoter signals. *J Mol Biol* **297:** (2) 335.

### 7 Pharmacogenomics

Alizadeh A, Eisen M, Davis RE, Ma C, Sabet H, Tran T, Powell JI, Yang L, Marti GE, Moore DT, Hudson JR, Chan WC, Greiner T, Weisenburger D, Armitage JO, Lossos I, Levy R, Botstein D, Brown PO, Staudt LM. 1999. NIH/NCI, Metab Branch, Div Clin Sci, Bethesda, Md 20892, USA. The lymphochip: A specialized cDNA microarray for the genomic-scale analysis of gene expression in normal and malignant lymphocytes. *Cold Spring Harb Symp Quant Biol* **64:** 71.

Bammert GF, Fostel JM*. 2000. *Pharmacia & Upjohn Inc, 7000 Portage Rd, Kalamazoo, In 49001, USA. Genome-wide expression patterns in *Saccharomyces cerevisiae*: Comparison of drug treatments and genetic alterations affecting biosynthesis of ergosterol. *Antimicrob Agents Chemother* **44:** (5) 1255.

Bartosiewicz M, Trounstine M, Barker D, Johnston R, Buckpitt A. 2000. Univ Calif, Dept Mol Biosci, Davis, Ca 95616, USA. Development of a toxicological gene array and quantitative assessment of this technology. *Arch Biochem Biophys* **376:** (1) 66.

Browne MJ. 2000. SmithKline Beecham, New Frontiers Sci Pk, 3rd Ave, Harlow CM19 5AW, England. Analysis of large gene databases for discovery of novel therapeutic agents. *J Biotechnol* **78:** (3) 247.

Burger H, Capello A, Schenk PW, Stoter G, Brouwer J, Nooter K*. 2000. *Univ Rotterdam Hosp, Dept Med Oncol, Rotterdam, The Netherlands. A genome-wide screening in *Saccharomyces cerevisiae* for genes that confer resistance to the anticancer agent cisplatin. *Biochem Biophys Res Commun* **269:** (3) 767.

Cohen P, Bouaboula M, Bellis M, Baron V, Jbilo O, Poinot-Chazel C, Galiegue S, Hadibi EH, Casellas P*. 2000. *371 rue Prof J Blayac, FR-34184 Montpellier 04, France. Monitoring cellular responses to *Listeria monocytogenes* with oligonucleotide arrays. *J Biol Chem* **275:** (15) 11181.

Dubinsky MC, Lamothe S, Yang HY, Targan SR, Sinnett D, Theoret Y, Seidman EG*. 2000. *Univ Montreal, Hop St Justine, Div Gastroenterol & Nutr, 3175 Cote St Catherine Rd, Montreal, Quebec, Canada H3T 1C5. Pharmacogenomics and metabolite measurement for 6-mercaptopurine therapy in inflammatory bowel disease. *Gastroenterology* **118:** (4) 705.

Eckmann L, Smith JR, Housley MP, Dwinell MB, Kagnoff MF. 2000. Univ Calif San Diego, Dept Med, 9500 Gilman Dr, La Jolla, Ca 92093, USA. Analysis by high density cDNA arrays of altered gene

Copyright © 2000 John Wiley & Sons, Ltd. 

expression in human intestinal epithelial cells in response to infection with the invasive enteric bacteria *Salmonella*. *J Biol Chem* **275:** (19) 14084.

Gray JW, Collins C. 2000. Univ Calif, Ctr Canc, 2340 Sutter St, San Francisco, Ca 94143, USA. Genome changes and gene expression in human solid tumors. *Carcinogenesis* **21:** (3) 443.

Gutierrez JA. 2000. Elanco Anim Hlth, 2001 West Main St, POB 708, Greenfield, In 46140, USA. Genomics: From novel genes to new therapeutics in parasitology. *Int J Parasitol* **30:** (3) 247.

Pennie WD, Tugwood JD, Oliver GJA, Kimber I. 2000. AstraZeneca, Cent Toxicol Lab, Alderley Pk, Macclesfield SK10 4TJ, England. The principles and practice of toxicogenomics: Applications and opportunities. *Toxicol Sci* **54:** (2) 277.

Schuster M, Wasserbauer E, Einhauer A, Ortner C, Jungbauer A*, Hammerschmid F, Werner G. 2000. *Novartis Forschungsinst, Vienna, Austria. Protein expression strategies for identification of novel target proteins. *J Biomol Screen* **5:** (2) 89.

Stoll M, Kwitek-Black AE, Cowley AW, Harris EL, Harrap SB, Krieger JE, Printz MP, Provoost AP, Sassard J, Jacob HJ*. 2000. *Med Coll Wisconsin, Dept Physiol, 8701 Watertown Plank Rd, Milwaukee, Wi 53226, USA. New target regions for human hypertension *via* comparative genomics. *Genome Res* **10:** (4) 473.

Strausberg RL, Buetow KH, Emmert-Buck MR, Klausner RD. 2000. NIH/NCI, Bethesda, Md 20892, USA. The cancer genome anatomy project: Building an annotated gene index. *Trends Genet* **16:** (3) 103.

Vediyappan G, Bikandi J, Braley R, Chaffin WL*. 2000. *Texas Tech Univ, Hlth Sci Ctr, Dept Immunol & Microbiol, Lubbock, Tx 79430, USA. Cell surface proteins of *Candida albicans*: Preparation of extracts and improved detection of proteins. *Electrophoresis* **21:** (5) 956.

### 8 Large-scale mutagenesis programmes

Anderson KV. 2000. Sloan Kettering Inst, Prog Mol Biol, 1275 York Ave, New York, NY 10021, USA. Finding the genes that direct mammalian development: ENU mutagenesis in the mouse. *Trends Genet* **16:** (3) 99.

Ding DQ, Tomita Y, Yamamoto A, Chikashige Y, Haraguchi T, Hiraoka Y*. 2000. *Communications Res Labs, Struct Biol Sect, Nishi ku, 588-2 Iwaoka cho, Kobe, Hyogo 651 2492, Japan. Large-scale screening of intracellular protein localization in living fission yeast cells by the use of a GFP-fusion genomic DNA library. *Genes Cells* **5:** (3) 169.

Dockendorff TC, Robertson SE, Faulkner DL, Jongens TA*. 2000. *Univ Penn, Dept Genet, 422 Curie Blvd, Philadelphia, Pa 19104, USA. Genetic characterization of the 44D-45B region of the *Drosophila melanogaster* genome based on an F2 lethal screen. *Mol Gen Genet* **263:** (1) 137.

Dubrova YE, Plumb M, Gutierrez B, Boulton E, Jeffreys AJ. 2000. Univ Leicester, Dept Genet, Univ Rd, Leicester LE1 7RH, England. Genome stability: Transgenerational mutation by radiation. *Nature* **405:** (6782) 37.

Lucau-Danila A, Wysocki R, Roganti T, Foury F*. 2000. *Univ Catholique Louvain, Unit Biochim Physiol, Pl Croix-du-Sud 2-20, BE-1348 Louvain-la-Neuve, Belgium. Systematic disruption of 456 ORFs in the yeast *Saccharomyces cerevisiae*. *Yeast* **16:** (6) 547.

McCallum CM, Comai L, Greene EA, Henikoff S*. 2000. *Fred Hutchinson Canc Res Ctr, Div Basic Sci, Seattle, Wa 98109, USA. Targeted screening for induced mutations. *Nat Biotechnol* **18:** (4) 455.

Vandenbol M, Fairhead C. 2000. Fac Univ Sci Agron Gembloux, Unite Microbiol, 6 Ave Marechal Juin, BE-5030 Gembloux, Belgium. Mass-murder deletion of 19 ORFs from *Saccharomyces cerevisiae* chromosome XI. *Gene* **247:** (1-2) 45.

### 9 Functional complementation

Heintz N. 2000. Rockefeller Univ, Howard Hughes Med Inst, Mol Biol Lab, 1230 York Ave, New York, NY 10021, USA. Analysis of mammalian central nervous system gene expression and function using bacterial artificial chromosome-mediated transgenesis. *Hum Mol Genet* **9:** (6) 937.

### 10 Transcriptomics

Aach J, Rindone W, Church GM*. 2000. *Harvard Univ, Sch Med, Dept Genet, Boston, Ma 02115, USA. Systematic management and analysis of yeast gene expression data. *Genome Res* **10:** (4) 431.

Abe H, Shimoda C*. 2000. *Osaka City Univ, Dept Biol, Sumiyoshi ku, Osaka 558 8585, Japan. Autoregulated expression of *Schizosaccharomyces pombe* meiosis-specific transcription factor Mei4 and a genome-wide search for its target genes. *Genetics* **154:** (4) 1497.

Anderson JSJ, Parker R*. 2000. *Univ Arizona, Howard Hughes Med Inst, Tucson, Az 85721, USA. Computational identification of *cis*-acting elements affecting post-transcriptional control of gene expression in *Saccharomyces cerevisiae*. *Nucleic Acids Res* **28:** (7) 1604.

Carels N, Bernardi G*. 2000. *Stn Zool A Dohrn, Lab Evoluz Mol, Villa Comunale, IT-80121 Naples, Italy. The compositional organization and the expression of the *Arabidopsis* genome. *FEBS Lett* **472:** (2-3) 302.

Davis CA, Grate L, Spingola M, Ares M*. 2000. *Univ Calif, Ctr Mol Biol DNA, Sinsheimer Labs 423, Santa Cruz, Ca 95064, USA. Test of intron predictions reveals novel splice sites, alternatively spliced mRNAs and new introns in meiotically regulated genes of yeast. *Nucleic Acids Res* **28:** (8) 1700.

De Risi J, Van den Hazel B, Marc P, Balzi E, Brown P, Jacq C, Goffeau A*. 2000. *Univ Catholique Louvain, Unite Biochim Physiol, Pl Croix Sud 2120, BE-1348 Louvain-la-Neuve, Belgium. Genome microarray analysis of transcriptional activation in multidrug resistance yeast mutants. *FEBS Lett* **470:** (2) 156.

Diehn M, Eisen MB, Botstein D, Brown PO*. 2000. *Stanford Univ, Sch Med, Dept Biochem, Stanford, Ca 94305, USA. Large-scale identification of secreted and membrane-associated gene products using DNA microarrays. *Nat Genet* **25:** (1) 58.

Gardiner K, Mural R, Wener T. 2000. Eleanor Roosevelt Inst Canc Res, 1899 Gaylord St, Denver, Co 80206, USA. Report of the IXth international workshop on the identification of transcribed sequences. *Cytogenet Cell Genet* **88:** (1-2) 2.

Hernandez A, Figueroa A, Rivas LA, Parro V, Mellado RP*. 2000. *CSIC, Ctr Nacl Biotecnol, Campus Univ Autonoma, Cantoblanco, ES-28049 Madrid, Spain. RT-PCR as a tool for systematic transcriptional analysis of large regions of the *Bacillus subtilis* genome. *Microbiology* **146:** (4) 823.

Shaw-Smith CJ, Coffey AJ, Huckle E, Durham J, Campbell EA, Freeman TC, Walters JRF*, Bentley DR. 2000. *Univ London, Imperial Coll Sci & Technol, Hammersmith Hosp, Sch Med, Gastroenterol Sect, London W12 0NN, England. Improved method for detecting differentially expressed genes using cDNA indexing. *Biotechniques* **28:** (5) 958.

Sudarsanam P, Iyer VR, Brown PO, Winston F*. 2000. *Stanford Univ, Dept Biochem, 300 Pasteur Ave, Stanford, Ca 94305, USA. Whole-genome expression analysis of *snf/swi* mutants of *Saccharomyces cerevisiae*. *Proc Natl Acad Sci U S A* **97:** (7) 3364.

Wang E, Miller LD, Ohnmacht GA, Liu ET, Marincola FM*. 2000. *NIH/NCI, Surg Branch, Div Clin Sci, Bethesda, Md 20892, USA. High-fidelity mRNA amplification for gene profiling. *Nat Biotechnol* **18:** (4) 457.

Yano N, Endoh M, Fadden K, Yamamshita H, Kane A, Sakai H, Rifai A*. 2000. *Brown Univ, Rhode Isl Hosp, Dept Pathol, 593 Eddy St, Providence, RI 02903, USA. Comprehensive gene expression profile of the adult human renal cortex: Analysis by cDNA array hybridization. *Kidney Int* **57:** (4) 1452.

Ye Z, Connor JR*. 2000. *Penn State Univ, Coll Med, Dept Anat & Neurosci, Milton S Hershey Med Ctr, POB 850, Hershey, Pa 17033, USA. Identification of iron responsive genes by screening cDNA libraries from suppression subtractive hybridization with antisense probes from three iron conditions. *Nucleic Acids Res* **28:** (8) 1802.

Yoshida K, Ishio I, Nakagawa E, Yamamoto Y, Yamamoto M, Fujita

Copyright © 2000 John Wiley & Sons, Ltd. 

Y*. 2000. *Fukuyama Univ, Dept Biotechnol, 985 Sanzo, Higashimura cho, Fukuyama, Hiroshima 729 02, Japan. Systematic study of the gene expression and transcription organization of the *gntZ-ywaA* region of the *Bacillus subtilis* genome. *Microbiology* **146:** (3) 573.

### 11 Proteomics

Chang WWP, Huang L, Shen M, Webster C, Burlingame AL, Roberts JKM*. 2000. *Univ Calif, Dept Biochem, Riverside, Ca 92521, USA. Patterns of protein synthesis and tolerance of anoxia in root tips of maize seedlings acclimated to a low-oxygen environment, and identification of proteins by mass spectrometry. *Plant Physiol* **122:** (2) 295.

Goodlett DR, Bruce JE, Anderson GA, Rist B, Pasa-Tolic L, Fiehn O, Smith RD, Aebersold R. 2000. Univ Washington, Dept Mol Biotechnol, Hlth Sci Bldg, Box 357730, Seattle, Wa 98195, USA. Protein identification with a single accurate mass of a cysteine-containing peptide and constrained database searching. *Anal Chem* **72:** (6) 1112.

Joubert R, Brignon P, Lehmann C, Monribot C, Gendre F, Boucherie H*. 2000. *Inst Biochim & Genet Cell, UPR CNRS 9026, 1 rue Camille Saint-Saens, FR-33077 Bordeaux, France. Two-dimensional gel analysis of the proteome of lager brewing yeasts. *Yeast* **16:** (6) 511.

Lai CH, Chou CY, Chang LY, Liu CS, Lin WC*. 2000. *Acad Sinica, Inst Biomed Sci, Taipei 115, Taiwan. Identification of novel human genes evolutionarily conserved in *Caenorhabditis elegans* by comparative proteomics. *Genome Res* **10:** (5) 703.

Molloy MP, Herbert BR, Slade MB, Rabilloud T, Nouwens AS, Williams KL, Gooley AA. 2000. Univ Michigan, Sch Med, Dept Biol Chem, Ann Arbor, Mi 48109, USA. Proteomic analysis of the *Escherichia coli* outer membrane. *Eur J Biochem* **267:** (10) 2871.

Perrin C, Gonzalez-Marquez H, Gaillard JL, Bracquart P*, Guimont C. 2000. *Univ Nancy 1, INRA, Lab Biosci Aliment, BP 239, FR-54506 Vandoeuvre-les-Nancy, France. Reference map of soluble proteins from *Streptococcus thermophilus* by two-dimensional electrophoresis. *Electrophoresis* **21:** (5) 949.

Rosenkrands I, Weldingh K, Jacobsen S, Hansen CV, Florio W, Gianetri I, Andersen P*. 2000. *Statens Serum Inst, Dept TB Immunol, 5 Artillerivej, DK-2300 Copenhagen S, Denmark. Mapping and identification of *Mycobacterium tuberculosis* proteins by two-dimensional gel electrophoresis, microsequencing and immunodetection. *Electrophoresis* **21:** (5) 935.

### 12 Protein structural genomics

Chakravarty S, Varadarajan R*. 2000. *Jawaharlal Nehru Ctr Adv Sci Res, Chem Biol Unit, IN-560004 Bangalore, India. Elucidation of determinants of protein stability through genome sequence analysis. *FEBS Lett* **470:** (1) 65.

McCraith S, Holtzman T, Moss B, Fields S*. 2000. *Univ Washington, Dept Genet, Howard Hughes Med Inst, Box 357360, Seattle, Wa 98195, USA. Genome-wide analysis of vaccinia virus protein-protein interactions. *Proc Natl Acad Sci U S A* **97:** (9) 4879.

Travers KJ, Patil CK, Wodicka L, Lockhart DJ, Weissman JS*, Walter P. 2000. *Univ Calif, Dept Cellular Pharmacol, San Francisco, Ca 94143, USA. Functional and genomic analyses reveal an essential coordination between the unfolded protein response and ER-associated degradation. *Cell* **101:** (3) 249.

Usuka J, Brendel V*. 2000. *Iowa State Univ Sci & Technol, Dept Zool & Genet, 2112 Mol Biol Bldg, Ames, Ia 50011, USA. Gene structure prediction by spliced alignment of genomic DNA with protein sequences: Increased accuracy by differential splice site scoring. *J Mol Biol* **297:** (5) 1075.

Wilson CA, Kreychman J, Gerstein M*. 2000. *Yale Univ, Dept Mol Biophys & Biochem, 266 Whitney Ave, POB 208114, New Haven, Ct 06520, USA. Assessing annotation transfer for genomics: Quantifying the relations between protein sequence structure and function through traditional and probabilistic scores. *J Mol Biol* **297:** (1) 233.

### 13 Metabolomics

Oh MK, Liao JC*. 2000. *UCLA, Dept Chem Engn, Los Angeles, Ca 90095, USA. Gene expression profiling by DNA microarrays and metabolic fluxes in *Escherichia coli*. *Biotechnol Prog* **16:** (2) 278.

Ouzounis CA, Karp PD. 2000. EMBL, European Bioinformat Inst, Cambridge Outstn, Computational Genomics Grp, Cambridge CB10 1SD, England. Global properties of the metabolic map of *Escherichia coli*. *Genome Res* **10:** (4) 568.

Schilling CH, Palsson BO. 2000. Univ Calif San Diego, Dept Bioengn, La Jolla, Ca 92093, USA. Assessment of the metabolic capabilities of *Haemophilus influenzae* Rd through a genome-scale pathway analysis. *J Theor Biol* **203:** (3) 249.

Selkov E, Overbeek R, Kogan Y, Chu L, Vonstein V, Holmes D, Silver S, Hasselkorn R, Fonstein M*. 2000. *Integrated Genomics, 2201 West Campbell Pk Dr, Chicago, Il 60612, USA. Functional analysis of gapped microbial genomes: Amino acid metabolism of *Thiobacillus ferrooxidans*. *Proc Natl Acad Sci U S A* **97:** (7) 3509.

### 14 Genomic approaches to development

Coller HA, Grandori C, Tamayo P, Colbert T, Lander ES, Eisenman RN, Golub TR. 2000. Fred Hutchinson Canc Res Ctr, 100 Fairview Ave Nth, Seattle, Wa 98109, USA. Expression analysis with oligonucleotide microarrays reveals that MYC regulates genes involved in growth, cell cycle, signaling, and adhesion. *Proc Natl Acad Sci U S A* **97:** (7) 3260.

Kelly DL, Rizzino A*. 2000. *Univ Nebraska, Eppley Inst Res Canc, Nebraska Med Ctr, Omaha, Ne 68198, USA. DNA microarray analyses of genes regulated during the differentiation of embryonic stem cells. *Mol Reprod Dev* **56:** (2) 113.

Rogge L, Bianchi E, Biffi M, Bono E, Chang SYP, Alexander H, Santini C, Ferrari G, Sinigaglia L, Seiler M, Neeb M, Mous J, Sinigaglia F, Certa U. 2000. Roche Milano Ricerche, Milan, Italy. Transcript imaging of the development of human T helper cells using oligonucleotide arrays. *Nat Genet* **25:** (1) 96.

Samach A, Onouchi H, Gold SE, Ditta GS, Schwarz-Sommer Z, Yanofsky MF, Coupland G*. 2000. *John Innes Ctr Plant Sci Res, Norwich NR4 7UH, England. Distinct roles of CONSTANS target genes in reproductive development of *Arabidopsis*. *Science* **288:** (5471) 1613.

Sehl PD, Tai JYN, Hillan KJ, Brown LA, Goddard A, Yang RH, Jin HK, Lowe DG*. 2000. *Genentech Inc, Dept Cardiovasc Res, Mail Stop 42, 1 DNA Way, Sth San Francisco, Ca 94080, USA. Application of cDNA microarrays in determining molecular phenotype in cardiac growth, development, and response to injury. *Circulation* **101:** (16) 1990.

### 15 Technological advances

Ahmadian A, Gharizadeh B, Gustafsson AC, Sterky F, Nyren P, Uhlen M, Lundeberg J*. 2000. *Royal Inst Technol, Dept Biotechnol, SE-10044 Stockholm, Sweden. Single-nucleotide polymorphism analysis by pyrosequencing. *Anal Biochem* **280:** (1) 103.

Baba Y. 2000. Address not available. Ultra high-performance DNA analysis by microchip technology. Integration of electrochemical detection. *Electrochemistry* **68:** (3) 197.

Borchers C, Peter JF, Hall MC, Kunkel TA, Tomer KB*. 2000. *NIH/Natl Inst Environm Hlth Sci, Struct Biol Lab, POB 12233, Res Triangle Park, NC 27709, USA. Identification of in-gel digested proteins by complementary peptide mass fingerprinting and tandem mass spectrometry data obtained on an electrospray ionization quadrupole time-of-flight mass spectrometer. *Anal Chem* **72:** (6) 1163.

Breaux GA, Green-Church KB, France A, Limbach PA*. 2000. *Louisiana State University, Department Chem, 232 Choppin Hall, Baton Rouge, La 70803, USA. Surfactant-aided, matrix-assisted laser desorption/ionization mass spectrometry of hydrophobic and hydrophilic pep-

Copyright © 2000 John Wiley & Sons, Ltd. 

tides. *Anal Chem* **72:** (6) 1169.

Carlisle AJ, Prabhu VV, Elkahloun A, Hudson J, Trent JM, Linehan WM, Williams ED, Emmert-Buck MR, Liotta LA, Munson PJ, Krizman DB*. 2000. *Adv Technol Ctr, 8717 Grovement Circle, Gaithersburg, Md 20877, USA. Development of a prostate cDNA microarray and statistical gene expression analysis package. *Mol Carcinog* **28:** (1) 12.

Chen X, Smith LM, Bradbury EM. 2000. Los Alamos Natl Lab, Div Chem Sci & Technol, Los Alamos, NM 87544, USA. Site-specific mass tagging with stable isotopes in proteins for accurate and efficient protein identification. *Anal Chem* **72:** (6) 1134.

Choe LH, Lee KH*. 2000. *Cornell Univ, 120 Olin Hall, Ithaca, NY 14853, USA. A comparison of three commercially available isoelectric focusing units for proteome analysis: The Multiphor, the IPGphor and the Protean IEF cell. *Electrophoresis* **21:** (5) 993.

Crabtree HJ, Bay SJ, Lewis DF, Zhang JZ, Coulson LD, Fitzpatrick GA, Delinger SL, Harrison DJ, Dovichi NJ*. 2000. *Univ Alberta, Dept Chem, Edmonton, Alberta, Canada T6G 2G2. Construction and evaluation of a capillary array DNA sequencer based on a micromachined sheath-flow curvette. *Electrophoresis* **21:** (7) 1329.

Deamer DW, Akeson M. 2000. Univ Calif, Dept Chem & Biochem, Santa Cruz, Ca 95064, USA. Nanopores and nucleic acids: Prospects for ultrarapid sequencing. *Trends Biotechnol* **18:** (4) 147.

Decraene C, Reguigne-Arnould I, Auffray C, Pietu G*. 1999. *CNRS ERS 1984, 19 rue Guy Moquet, BP 8, FR-94801 Villejuif, France. Reverse transcription in the presence of dideoxynucleotides to increase the sensitivity of expression monitoring with cDNA arrays. *Biotechniques* **27:** (5) 962.

Gevaert K, Eggermont L, Demol H, Vandekerckhove J. 2000. State Univ Ghent, Flanders Interuniv Inst Biotechnol, Fac Med, Dept Med Prot Res, Kl Ledeganckstr 35, BE-9000 Ghent, Belgium. A fast and convenient MALDI-MS based proteomic approach: Identification of components scaffolded by the actin cytoskeleton of activated human thrombocytes. *J Biotechnol* **78:** (3) 259.

Gong BW, Ge RW*. 2000. *Natl Univ Singapore, Fac Sci, Dept Biol Sci, 10 Kent Ridge Crescent, SG-119260 Singapore, Rep Singapore. Using the SMART™ cDNA system to map the transcription intitiation site. *Biotechniques* **28:** (5) 846.

Han J, Craighead HG*. 2000. *Cornell Univ, Sch Appl & Engn Phys, Ithaca, NY 14853, USA. Separation of long DNA molecules in a microfabricated entropic trap array. *Science* **288:** (5468) 1026.

Hanafi-Bagby D, Piunno PAE, Wust CC, Krull UJ*. 2000. *Univ Toronto, Chem Sensors Grp, 3359 Mississauga Rd Nth, Mississauga, Ontario, Canada L5L 1C6. Concentration dependence of a thiazole orange derivative that is used to determine nucleic acid hybridization by an optical biosensor. *Anal Chim Acta* **411:** (1-2) 19.

Hoevel T, Holz H, Kubbies M. 1999. Roche Pharmaceut Res, Dept Cell Analyt, Nonnenwald 2, DE-82377 Penzberg, Germany. Cisplatin-digoxigenin mRNA labeling for nonradioactive detection of mRNA hybridized onto nucleic acid cDNA arrays. *Biotechniques* **27:** (5) 1064.

Jackson JA, Matthews D. 2000. Natl Inst Agr Bot, Huntingdon Rd, Cambridge CB3 0LE, England. Modified inter-simple sequence repeat PCR protocol for use in conjunction with the Li-cor gene ImagIR$_2$ DNA analyzer. *Biotechniques* **28:** (5) 914.

Jensen PK, Pasa-Tolic L, Peden KK, Martinovic S, Lipton MS, Anderson GA, Tolic N, Wong KK, Smith RD*. 2000. *Pacific NW Labs, Environm Mol Sci Lab, POB 999, Mail Stop K8-98, Richland, Wa 99352, USA. Mass spectrometric detection for capillary isoelectric focusing separations of complex protein mixtures. *Electrophoresis* **21:** (7) 1372.

Kell DB, King RD. 2000. Univ Coll Wales, Inst Biol Sci, Aberystwyth SY23 3DD, Wales. On the optimization of classes for the assignment of unidentified reading frames in functional genomics programmes: The need for machine learning. *Trends Biotechnol* **18:** (3) 93.

Knoblauch M, Gosele C, Zimdahl H, Hieke B, Himmelbauer H, Schalkwyk L, Lindpaintner K, Ganten D, Lehrach H. 2000. Max Delbruck Ctr Mol Med, Robert Rossle Str 10, DE-13122 Berlin, Germany. New tools for the high throughput characterization of rat genomic DNA samples. *J Exp Anim Sci* **41:** (1-2) 35.

Kotova EY, Kreindlin EY, Barsky VE, Mirzabekov AD. 2000. Russian Acad Sci, VA Engelhardt Mol Biol Inst, RU-117984 Moscow, Russia. Effects of various fluorochromes and competition between labeled oligonucleotides on their hybridization to oligonucleotides immobilized on biological microchips. *Mol Biol-Engl Tr* **34:** (2) 207.

Lazowski KW, Kaczmarek L. 2000. M Nencki Inst Expt Biol, Mol Neurobiol Lab, 3 Pasteur St, PL-02093 Warsaw, Poland. Highly sensitive detection of hybridization of oligonucleotides to specific sequences of nucleic acids by application of fluorescence resonance energy transfer. *Antisense Nucleic Acid Drug Dev* **10:** (2) 97.

Le Coutre J, Whitelegge JP, Gross A, Turk E, Wright EM, Kaback HR, Faull KF. 2000. UCLA, Howard Hughes Med Inst, Dept Phys, Box 951662, Los Angeles, Ca 90095, USA. Proteomics on full-length membrane proteins using mass spectrometry. *Biochemistry* **39:** (15) 4237.

Li LJ, Garden RW, Sweedler JV. 2000. Univ Illinois, Dept Chem, 1209 West Calif St, Urbana, Il 61801, USA. Single-cell MALDI: A new tool for direct peptide profiling. *Trends Biotechnol* **18:** (4) 151.

Maire E, Lelievre E, Brau D, Lyons A, Woodward M, Fafeur V, Vandenbunder B*. 2000. *Inst Pasteur, Inst Biol, CNRS EP 560, 1 rue Calmette, BP 447, FR-59021 Lille, France. Development of an ultralow-light-level luminescence image analysis system for dynamic measurements of transcriptional activity in living and migrating cells. *Anal Biochem* **280:** (1) 118.

Matsuhara S, Jingu F, Takahashi T*, Komeda Y. 2000. *Hokkaido Univ, Grad Sch Sci, Div Biol Sci, N10 W8, Sapporo, Hokkaido 060 081, Japan. Heat-shock tagging: A simple method for expression and isolation of plant genome DNA flanked by T-DNA insertions. *Plant J* **22:** (1) 79.

Okamoto T, Suzuki T, Yamamoto N*. 2000. *Canon Res Ctr, 5-1 Morinosato Wakamiya, Atsugi, Kanagawa 243 019, Japan. Microarray fabrication with covalent attachment of DNA using Bubble Jet technology. *Nat Biotechnol* **18:** (4) 438.

Sakiyama T, Takami H*, Ogasawara N, Kuhara S, Kozuki T, Doga K, Ohyama A, Horikoshi K. 2000. *Japan Marine Sci & Technol Ctr, Deep Sea Microogranisms Res Grp, 2-15 Natsushima cho, Kanagawa 237 0061, Japan. An automated system for genome analysis to support microbial whole-genome shotgun sequencing. *Biosci Biotechnol Biochem* **64:** (3) 670.

Schriml LM, Peterson RJ, Gerrard B, Dean M*. 2000. *NCI, Frederick Canc Res & Dev Ctr, Bldg 560, Frederick, Md 21702, USA. Use of denaturing HPLC to map human and murine genes and to validate single-nucleotide polymorphisms. *Biotechniques* **28:** (4) 740.

Shevchenko A, Loboda A, Shevchenko A, Ens W, Standing KG. 2000. European Mol Biol Lab, Peptide & Prot Grp, Meyerhofstr 1, DE-69012 Heidelberg, Germany. MALDI quadrupole time-of-flight mass spectrometry: A powerful tool for proteomic research. *Anal Chem* **72:** (9) 2132.

Spencer WE, Christensen MJ*. 1999. *Brigham Young Univ, Dept Food Sci & Nutr, Provo, Ut 84602, USA. Multiplex relative RT-PCR method for verification of differential gene expression. *Biotechniques* **27:** (5) 1044.

Stuebe ET, Steward JQ, Chinwalla A, Cook LL, Cook M, Fronick B, Miller K, Mullen MK, O'Brien D, Panussis DA, Pohl C, Snider JE, Strong J, Williams D, WIlson RK, Tibbetts C, Mardis ER*. 2000. *Washington Univ, Genome Sequencing Ctr, 4444 Forst Pk Blvd, St Louis, Mo 63108, USA. Modification of a commercially available DNA sequencer to increase sample throughput. *IEEE Eng Med Biol Mag* **19:** (2) 101.

Thomas JJ, Bakhtiar R, Siuzdak G*. 2000. *Scripps Res Inst, Beckman Ctr Chem Sci, 10550 Nth Torrey Pines Rd, La Jolla, Ca 92037, USA. Mass spectrometry in viral proteomics. *Account Chem Res* **33:** (3) 179.

Unger KK, Racaityte K, Wagner K, Miliotis T, Edholm LE, Bischoff R, Marko-Varga G. 2000. Univ Mainz, Inst Anorgan Chem & Analyt Chem, Duesbergweg 10-14, DE-550099 Mainz, Germany. Is multidimensional high performance liquid chromatography (HPLC) an alternative in protein analysis to 2D gel electrophoresis? *J High Res Chromatogr* **23:** (3) 259.

Wall DB, Kachman MT, Gong SY, Hinderer R, Parus S, Misek DE, Ha-

Copyright © 2000 John Wiley & Sons, Ltd. 

nash SM, Lubman DM*. 2000. *Univ Michigan, Dept Chem, Ann Arbor, Mi 48109, USA. Isoelectric focusing nonporous RP-HPLC: A two-dimensional liquid-phase separation method for mapping of cellular proteins with identification using MALDI-TOF mass spectrometry. *Anal Chem* **72:** (6) 1099.

Weigel D, Ahn JH, Blazquez MA, Borevitz JO, Christensen SK, Fankhauser C, Ferrandiz C, Kardailsky I, Malancharuvil EJ, Neff MM, Nguyen JT, Sato S, Wang ZY, Xia Y, Dixon RA, Harrison MJ, Lamb CJ, Yanofsky MF, Chory J. 2000. Salk Inst Biol Studies, Plant Biol Lab, 10010 Nth Torrey Pines Rd, La Jolla, Ca 92037, USA. Activation tagging in *Arabidopsis*. *Plant Physiol* **122:** (4) 1003.

Wright GL, Cazares LH, Leung SM, Nasim S, Adam BL, Yip TT, Schellhammer PF, Gong L, Vlahou A. 1999. Eastern Virginia Med Sch, Dept Microbiol & Mol Cell Biol, 700 West Olney Rd, Norfolk, Va 23501, USA. Proteinchip® surface enhanced laser desorption/ionization (SELDI) mass spectrometry: A novel protein biochip technology for detection of prostate cancer biomarkers in complex protein mixtures. *Prostate Cancer Prostatic Dis* **2:** (5-6) 264.

Xu YC, Bruch RC, Soper SA*. 2000. *Louisiana State Univ, Dept Chem, Baton Rouge, La 70803, USA. Microcapillary reactors using solid-phase DNA sequencing for direct sample introduction into slab gels. *Biotechniques* **28:** (5) 904.

Yasuda K, Okano K, Ishiwata S. 2000. Univ Tokyo, Grad Sch Arts & Sci, Dept Life Sci, Meguro ku, 3-8-1 Komaba, Tokyo 153 8902, Japan. Focal extraction of surface-bound DNA from a microchip using photo-thermal denaturation. *Biotechniques* **28:** (5) 1006.

Zammatteo N, Jeanmart L, Hamels S, Courtois S, Louette P, Hevesi L, Remacle J. 2000. Fac Univ Notre-Dame de la Paix, Lab Biochim Cellulaire, rue Bruxelles 61, BE-5000 Namur, Belgium. Comparison between different strategies of covalent attachment of DNA to glass surfaces to build DNA microarrays. *Anal Biochem* **280:** (1) 143.

Zheng HL, Yan W, Toppari J, Harkonen P*. 2000. *Univ Turku, Dept Astron, Kiinamyllynkatu 10, FI-20520 Turku, Finland. Improved non-radioactive RT-PCR method for relative quantification of mRNA. *Biotechniques* **28:** (5) 832.

### 16 Bioinformatics

Beitz E. 2000. Univ Tubingen, Fac Chem & Pharm, Morgenstelle 8, DE-72076 Tubingen, Germany. TeXshade: Shading and labeling of multiple sequence alignments using LaTeX2e. *Bioinformatics* **16:** (2) 135.

Birney E, Durbin R. 2000. Sanger Ctr, Wellcome Trust Genome Campus, Cambridge CB10 1SA, England. Using GeneWise in the *Drosophila* annotation experiment. *Genome Res* **10:** (4) 547.

Bouck J, McLeod MP, Worley K, Gibbs RA. 2000. Baylor Coll Med, Dept Mol & Human Genet, Human Genome Sequencing Ctr, Room N1521, Mail Stop BCM226, Houston, Tx 77030, USA. The Human Transcript Database: A catalogue of full length cDNA inserts. *Bioinformatics* **16:** (2) 176.

Bussow K, Nordhoff E, Lubbert C, Lehrach H, Walter G. 2000. Max Planck Inst Mol Genet, Ihnestr 73, DE-14195 Berlin, Germany. A human cDNA library for high-throughput protein expression screening. *Genomics* **65:** (1) 1.

Cuff JA, Birney E, Clamp ME, Barton GJ*. 2000. *EMBL Outstn, European Bioinformat Inst, Wellcome Trust Genome Campus, Cambridge CB10 1SD, England. ProtEST: Protein multiple sequence alignments from expressed sequence tags. *Bioinformatics* **16:** (2) 111.

Doolittle RF. 2000. Univ Calif San Diego, Ctr Mol Genet, La Jolla, Ca 92093, USA. On the trail of protein sequences. *Bioinformatics* **16:** (1) 24.

Getz G, Levine E, Domany E, Zhang MQ*. 2000. *Cold Spring Harbor Lab, POB 100, Cold Spring Harbor, NY 11724, USA. Super-parametric clustering of yeast gene expression profiles. *Physica A* **279:** (1-4) 457.

Henikoff JG, Henikoff S. 2000. Howard Hughes Med Inst, Seattle, Wa 98109, USA. *Drosophila* genomic sequence annotation using the BLOCKS plus database. *Genome Res* **10:** (4) 543.

Hodgman TC. 2000. Glaxo Wellcome Res & Dev Ltd, Med Res Ctr, Stevenage SG1 2NY, England. A historical perspective on gene/protein functional assignment. *Bioinformatics* **16:** (1) 10.

Junker V, Contrino S, Fleischmann W, Hermjakob H, Lang F, Magrane M, Martin MJ, Mitaritonna N, O'Donovan C, Apweiler R. 2000. EMBL Outstn, European Bioinformat Inst, Wellcome Trust Genome Campus, Hinxton CB10 1SD, England. The role Swiss-Prot and TrEMBL play in the genome research environment. *J Biotechnol* **78:** (3) 221.

Krogh A. 2000. Tech Univ Denmark, Ctr Biol Sequence Anal, DK-2800 Lyngby, Denmark. Using database matches with HMMgene for automated gene detection in *Drosophila*. *Genome Res* **10:** (4) 523.

Linder P, Gasteiger E, Bairoch A. 2000. Ctr Med Univ, Dept Biochim Med, 1 rue Michel Servet, CH-1211 Geneve 4, Switzerland. A comprehensive web resource on RNA helicases from the baker's yeast *Saccharomyces cerevisiae*. *Yeast* **16:** (6) 507.

McGuire G, Wright F*. 2000. *Scottish Crop Res Inst, Dundee DD2 5DA, Scotland. TOPAL 2.0: Improved detection of mosaic sequences within multiple alignments. *Bioinformatics* **16:** (2) 130.

Mironov AA, Vinokurova NP, Gelfand MS. 2000. State Res Ctr Gosniigenet, RU-113545 Moscow, Russia. Software for analysis of bacterial genomes. *Mol Biol-Engl Tr* **34:** (2) 222.

Ohler U. 2000. Univ Erlangen Nurnberg, Lehrstuhl Mustererkennung, DE-91058 Erlangen, Germany. Promoter prediction on a genomic scale: The Adh experience. *Genome Res* **10:** (4) 539.

Panina EM, Mironov AA, Gelfand MS. 2000. State Res Ctr Gosniigenet, RU-113545 Moscow, Russia. Statistical analysis of complete bacterial genomes: Avoidance of palindromes and restriction-modification systems. *Mol Biol-Engl Tr* **34:** (2) 215.

Park J, Holm L, Chothia C. 2000. EMBL Outstation, European Bioinformat Inst, Cambridge CB10 1SD, England. Sequence search algorithm assessment and testing toolkit (SAT). *Bioinformatics* **16:** (2) 104.

Parra G, Blanco E, Guigo R*. 2000. *Univ Pompeu Fabra, IMIM, Grp Recerca Informat Med, ES-08003 Barcelona, Spain. GeneID in *Drosophila*. *Genome Res* **10:** (4) 511.

Purdom PW, Bradford PG, Tamura K, Kumar S. 2000. Indiana Univ, Dept Comp Sci, Bloomington, In 47405, USA. Single column discrepancy and dynamic max-mini optimizations for quickly finding the most parsimonious evolutionary trees. *Bioinformatics* **16:** (2) 140.

Reese MG, Hartzell G, Harris NL, Ohler U, Abril JF, Lewis SE. 2000. University California, Dept Mol & Cell Biol, Berkeley Drosophila Genome Project, 229 Stanley Hall, Berkeley, Ca 94720, USA. Genome annotation assessment in *Drosophila melanogaster*. *Genome Res* **10:** (4) 483.

Reese MG, Kulp D, Tammana H, Haussler D. 2000. Address as above. Genie: Gene finding in *Drosophila melanogaster*. *Genome Res* **10:** (4) 529.

Sahnoun I, Dehais P, Van Montagu M, Rossignol M, Rouze P*. 2000. *State Univ Ghent, Flanders Interuniv Inst Biotechnol, Dept Plant Genet, INRA, Kl Ledeganckstr 35, BE-9000 Ghent, Belgium. PPMdb: A plant plasma membrane database. *J Biotechnol* **78:** (3) 235.

Salamov AA, Solovyev VV*. 2000. *Sanger Ctr, Cambridge CB10 1SA, England. *Ab initio* gene finding in *Drosophila* genomic DNA. *Genome Res* **10:** (4) 516.

Sato N. 2000. Saitama Univ, Fac Sci, Dept Biochem & Mol Biol, 255 Shimoohkubo, Urawa, Saitama 338 8570, Japan. SISEQ: Manipulation of multiple sequence and large database files for common platforms. *Bioinformatics* **16:** (2) 180.

Schwartz S, Zhang Z, Frazer KA, Smit A, Riemer C, Bouck J, Gibbs R, Hardison R, Miller W*. 2000. *Penn State University, Department Comp Sci & Engn, University Park, Pa 16802, USA. PipMaker: A Web server for aligning two genomic DNA sequences. *Genome Res* **10:** (4) 577.

Tristem M. 2000. Univ London, Imperial Coll Sci Technol & Med, Dept Biol, Silkwood Pk, Ascot SL5 7PY, England. Identification and characterization of novel human endogenous retrovirus families by phylogenetic screening of the Human Genome Mapping Project database. *J Virol* **74:** (8) 3715.

Copyright © 2000 John Wiley & Sons, Ltd. 
